# Public transport pricing incentive schemes in a competitive market

**DOI:** 10.1371/journal.pone.0313439

**Published:** 2024-11-08

**Authors:** Lishuang Bian, Qizhou Hu, Minjia Tan

**Affiliations:** 1 School of Automation, Nanjing University of Science and Technology, Nanjing, China; 2 Ocean College, Jiangsu University of Science and Technology, Zhenjiang, China; Chang’an University, CHINA

## Abstract

In many countries, public transport (PT) services are offered by multiple operators with different modes, such as trains, metros, and buses, which are interchangeable across a single journey. This paper presents a model with one origin (O)-destination(D) path operated by two operators, each of which is responsible for different parts of the OD journey. One operator competes with potential third-party transport companies by offering discount incentives. Such an abstract settings has not been discussed in the literature. We explore how prices, demand, profits, and social welfare change with discounts through a theoretical analysis and numerical simulations under five scenarios. The results indicate that in all the scenarios the operator offering a discount incentive can always attract more passengers and increase its profits. Moreover, reducing the service time of operators offering discounts contributes to an increase in social welfare. Notably, this paper deduces for the first time that the demand scenario aimed at maximizing social welfare is twice as high as that aimed at maximizing total profit. However, in the scenario of maximum social welfare, the profitability of operators becomes challenging.

## 1. Introduction

In an increasingly competitive market, public transportation systems face the challenge of attracting and maintaining passengers. One fact is that some countries and urban areas have transformed some or all of their public transport systems into competitive mechanisms (e.g., the European passenger railway market [1], Seoul, Unums, Singapore, and the Czech Republic [2]); therefore, the duopoly competition [3–6] in economics can be applied to the field of transportation. In recent years, an increasing number of studies have reported that transportation markets are also natural monopoly markets and that operators benefit from large-scale transportation services. For example, Kurosaki and Singh [7] compared three models for introducing competition in rail freight transport in three countries and reported that intramodal competition of one type may not apply to another country. Ivaldi and Vibes [8,9] analysed inter and intramodal competition in the railway transport industry. Wong and Hensher [10] considered competition and ownership in land passenger transport. D’Alfonso et al. [11] modelled the effects of air transport and high-speed rail competition on the environment. Guihéry [12] investigated the opening to competition in Germany’s regional railway transport. However, operators of transportation face not only direct competition but also indirect competition from private cars, bicycles, taxis, and shuttle buses. Therefore, we establish a passenger travel transfer model that includes three modes of transportation and study the competition between one public transportation operator and potential third parties such as private cars, after the transfer point.

Pricing incentive schemes, are an important means to increase the attractiveness of public transportation and promote the use of public transit by adjusting passenger travel choices through economic incentives [13,14]. The pricing strategy for public transportation is usually based on an understanding of passenger travel behaviour. In a competitive market, operators need to consider the sensitivity of passengers to prices and their competitive relationship with other modes of transportation. Early research focused on fare elasticity and cost recovery [15–18], whereas in recent years, researchers have begun to focus on how to increase the market share of public transportation through incentive measures [19,20].

Public transport incentive strategies include but are not limited to discounts, ticket prices, periodic pricing, loyalty rewards, and demand-based dynamic pricing. For example, Li et al. [21] explored the factors influencing changes in travel behaviour via the adaptive stacked limit gradient boosting model and reported that gender, work status, preferred travel methods, and reward categories significantly influence user motivation Han et al. [22] proposed an incentive strategy that rewards passengers for transferring to other passengers’ boarding and alighting points, reducing detours and shortening the travel time of passengers. Li et al. [23] focused on personalised travel incentives, demand-responsive passenger incentives in transportation systems, and electric vehicle incentive policies. Niu and Clark [24] adopted the concept of differential privacy and proposed an incentive design to protect privacy for passengers transitioning from private cars to public transportation. He and Guan [25] designed government subsidy incentive contracts and logistics alliance payment incentive contracts to improve the quality of passenger and cargo services.

As a policy incentive, discounts are widely studied and applied [26,27]. For example, Beijing transit agencies implemented a discount pricing measure in 2016 that provided a 30% discount for passengers who checked in before 7:00 a.m. Zou et al. [28] measured the response of passengers to the rescheduling of discounted fares before peak hours by tracking smart card data and optimising the fares. In fact, in the above two examples, discounts are used as a necessary means to alleviate traffic congestion. Although some studies have investigated transfer fare discounts, Wey et al. [29] evaluated the effects of environmental factors and a transfer fare discount policy on the performance of an urban metro system. Li et al. [30] analysed the impact of fare discounts on passenger volume in Xiamen, China. However, the above studies focused on the impact of fare discounts or even transfer fare discounts only on the passenger flow of one transportation mode, without considering the effects of fare discounts under a competing market.

In the public transport market, researchers are committed to analysing and optimising the competitive relationships and pricing strategies of multimodal public transportation systems. For example, Pei and Wang [31] revealed the characteristics of competition and cooperation in urban public transportation systems in cold region cities by constructing a multimodal public transit Lotka–Volterra competition model. Hu and colleagues [32] focused on optimising high-speed railway ticket pricing, considering a comprehensive competitive environment with the aviation industry, and used a mixed integer linear programming model to balance the travel costs for passengers and the revenue for railway enterprises. Tan and colleagues [33] studied the pricing issue between demand-responsive transit and bike-sharing systems from a game-theory perspective, and proposed a nested two-layer planning pricing model. Pilar Socorro and Fernanda Viecens [34] studied the social and environmental effects of airline and high- speed train (HST) integration. However, most studies have investigated the competition between two types of public transportation without considering the impact of discount incentives on passengers’ transfer choices. Based on the model construction from references [5,6], this paper develops a passenger travel model that includes three transportation modes, highlighting the complementary relationship between two public transportation operators, with one of them competing with the potential 3^rd^ party by implementing discount incentives. Table 1 summarizes relevant studies of competitive model on public transport competition.

**Table 1:** Summary of the relevant studies on public transport competition.

Publication	Number of operators	Transfer point	Objective	Price incentive
Pei and Wang [31]	3	No	Reveal the characteristics of competition and cooperation	No
Hu et al. [20]	2	No	Minimize overall travel costs	No
Tan et al. [33]	2	No	Establish a feeder program	No
Socorro and Fernanda Viecens [34]	2	No	Analyse the social and environmental effects of airline and HST integration.	No
Wang et al.[35]	2	No	Analyse the influence of speed and frequency on social welfare	No
Clark et al. [20]	2	No	Analyse the influence of distance on quantity and price	No
This paper	3	Yes	Analyse the influence of discount incentives on profits, social welfare, quantity, and price	Yes

The main insights of this paper are follows. First, we propose a multimodal public transit competition model. Second, we derive the equilibria under five scenarios and focus on equilibrium fares, the number of passengers, profits, social welfare, and how they develop concerning discounts under different types of competition. We also study how the competition model of operators affects travel options, price, profits, and social welfare. Moreover, the impact of changes in travel time on the optimal profits and social welfare will also be studied.

The rest of the paper is organized as follows. Section 2 presents the model and accounts for central assumptions. Section 3 derives the market equilibrium under five competitive scenarios. Section 4 presents nine key theorems that clarify the dynamics of demand, fare, social welfare, and profitability across various scenarios. Section 5 conducts numerical simulations to validate the theorem results from Section 4. Finally, conclusions and implications are presented in Section 6.

## 2. Model development

In this section, we will articulate the model’s framework and elucidate the pertinent assumptions that underpin it. We will define consumer surplus and social welfare based on the model and proceed to derive the inverse demand function. Additionally, we will expound on the operators’ profit and cost structures. Finally, we will present an analysis of two distinct transfer selection outcomes that passengers may encounter during travel in the basic case scenario.

### 2.1. Problem description

**Fig 1:**
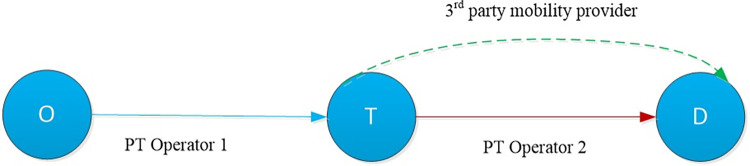
Illustrative network: One corridor with one compulsory transfer.

We consider a transportation corridor connecting an origin‒destination (OD) pair, as shown in Fig 1. There is one compulsory transfer, *T*, for travellers commuting between nodes *O* and *D*. Correspondingly, one trip is naturally divided into two legs: the leg before the transfer station and the one after. Two public transport (PT) operators serve the two legs. Thus, to a certain extent, the services provided by the two operators are complementary. PT Operator 1 serves exclusively between the origin and the transfer station. In contrast, there exists another 3^rd^ party offers a competing connection between transfer station *T* and destination *D* together with PT Operator 2. This abstract setting represents real practical situations, where the first and second legs are, respectively, the trunk and local (last mile) services, and different operators are providing the corresponding services. For example, in the Copenhagen metropolitan area, the two legs could be the Metro and bus company (e.g., Movia). Moreover, instead of using conventional buses, other mobility services, such as shared bikes, e-scooters, and car sharing, are also available for travellers completing last-mile trips.

Given the abovementioned setting, the following assumptions are made to facilitate our analysis.

**Assumption 1:** The travel demand (the number of passengers) is elastic, and depends on the generalized travel cost which includes the travel times, transit fare, and travel distance.

**Assumption 2:** We consider a simple distance-based ticket price scheme adopted by the two operators, meaning that the transit fare is a linear function concerning the distance. Distance-based fare discount schemes are also reflected in the existing literature [18].

**Assumption 3:** For the convenience of calculations, this paper defines the generalized travel costs associated with the 3^rd^ service provider as a constant.

**Assumption 4:** The travel time of PT Operator 2 is longer than that of the 3^rd^ party.

Under the aforementioned assumptions, we will delve into an elementary price incentive scheme, specifically examining a discount provision for transfer travellers who opt for the services of PT Operator 2. Our investigation is aimed at assessing the impact of this incentive mechanism on key performance metrics, including PT ridership, social welfare, and the profitability of operators.

### 2.2. Formulation of the base scenario

In this subsection, we will outline the two posttransfer station options available to passengers and present an illustration of a base scenario. Within the fundamental case, we posit that the two operators have established their ticket pricing strategies. Subsequently, PT Operator 2 will endeavour to introduce discounts aimed at enticing a larger share of passengers from third-party services. To conclude this segment, we will articulate three foundational theorems that pertain to the basic case, offering insights into the strategic interplay between operators and the implications for passenger behaviour and market outcomes.

#### 2.2.1. Demand side

First, let us define passengers’ choices. There are two options for passengers travelling from the origin to the destination. They are differentiated by the choice after the transfer station. Passengers can transfer either via PT Operator 2 or the 3^rd^ party service. For clarity, the two options are explicitly stated below, as they will be mentioned quite often in this paper.

*Option 1*: After arriving at the transfer station via PT Operator 1’s service, passengers cease using the PT service and select the 3^rd^ party’s service to travel to their destination.

*Option 2*: After arriving at the transfer station via PT Operator 1’s service, passengers select PT Operator 2’s service to travel to their destination.

We define passengers’ generalized travel cost associated with the two options as follows:

$$G_{1}=p_{1}+bt_{1}+K,$$ (1)

$$G_{2}=p_{1}+p_{2}+bt_{1}+bt_{2}-H(\lambda ,t_{1},t_{2})$$ (2)

where *G*_*i*_,*∀*_*i*_∈{1,2} is the generalised travel costs of passengers for Option *i*, *p*_*i*_ denotes the transit fare charged by PT Operator *i*; *t*_*i*_ is for the average travel time; *b* is the passenger’s value of the travel time used to convert the travel time into the equivalent monetary cost; and *K* is the generalized travel cost associated with the 3^rd^ service provider. Set *H*(*λ*,*t*_1_,*t*_2_) is the fare discounts offered by PT Operator 2 to attract passengers to use PT service. For simplicity, this study postulates that the discount depends on a discount ratio to be determined, and the operating distance of the two operators, represented by *t*_1_ and *t*_2_. For example, if Operator 1 covers a longer distance for the whole trip, then a lower discount is given to the travellers. In this study, the following function is proposed to compute the discount value.

$$H(\lambda ,t_{1},t_{2})=\lambda (t_{1}-t_{2}),$$ (3)

where *λ* = 0.05⋅(*i*+1) and the range of *i* is (0,30).

The representative consumer surplus (*CS*) refers to the difference between the highest fare of transportation services that passengers are willing to pay (passengers’ utility) and the actual fare of transportation services. Then, following Singh and Vives [3] the representative consumer surplus to be maximized is given by

$$CS=U(x_{1},x_{2})-G_{1}x_{1}-G_{2}x_{2},$$ (4)

where *x*_1_ and *x*_2_ denote the number of passengers travelling via options 1 and 2, respectively. *G*_*i*_*x*_*i*_,∀*i* = 1,2 gives the cost to travellers and *U*(*x*_1_,*x*_2_) computes passengers’ utility.

To define passengers’ utility, we adopt an approach similar to that of Clark et al. [19,20,36]. In their works, a strictly inverse demand function is specified on the basis of based Singh and Vives [3], and the two options are considered to be two products offered by two operators. Then, under the condition that passengers maximize their utility according to the quantities for the services provided by the two firms, the aggregated utility function is given by

$$U(x_{1},x_{2})=a_{1}x_{1}+a_{2}x_{2}-\frac{mx_{1}^{2}+2gx_{1}x_{2}+mx_{2}^{2}}{2}$$ (5)

where *a*_1_, *a*_2_, and *m* are positive, and where *m*^2^−*g*^2^>0, *a*_*i*_*m*−*a*_*j*_*g*>0 for *i*≠*j*, *i* = 1,2. *g*∈(0,1) measures the degree of complementarity between the services offered by the two operators according to Singh and Vives [3].

By maximizing the consumption surplus defined in Eq (4), we can obtain the inverse demands from the first-order condition as follows:

$$p_{1}=-mx_{1}-gx_{2}-W$$ (6)

$$p_{2}=(m-g)(x_{1}-x_{2})+R+H(\lambda ,t_{1},t_{2})$$ (7)

where $R=a_{2}-a_{1}-bt_{2}+K,$ $W=-a_{1}+bt_{1}+K$. Then, from Eqs (6) and (7), the direct demand for the services of the two operators can be obtained by

$$x_{1}=\frac{1}{g^{2}-m^{2}}[m(p_{1}+W)-g(p_{1}+p_{2}+W-H(\lambda ,t_{1},t_{2})-R)]$$ (8)

$$x_{2}=\frac{1}{g^{2}-m^{2}}[m(p_{1}+p_{2}+W-H(\lambda ,t_{1},t_{2})-R)-g(p_{1}+W)]$$ (9)

Then the total consumption surplus defined in Eq (4) can be expressed as:

$$CS(x_{1},x_{2})=\frac{mx_{1}^{2}+2gx_{1}x_{2}+mx_{2}^{2}}{2}$$ (10)

By substituting Eqs (8) and (9) into Eq (10) and rearranging the equation, we obtaine the following expression for consumer surplus as a function of price.


$$\begin{array}{l} CS(p_{1},p_{2})=\frac{m[{\left(p_{1}+W\right)}^{2}+{\left(p_{1}+p_{2}+W-H(\lambda ,t_{1},t_{2})-R\right]}^{2})}{2(m^{2}-g^{2})}\\ \;-\frac{-2g(p_{1}+p_{2}+W-H(\lambda ,t_{1},t_{2})-R)(p_{1}+W)}{2(m^{2}-g^{2})} \end{array}$$ (11)

#### 2.2.2. Supply side

Denote *y*_*i*_ as the number of passengers using PT Operator *i*’s service. According to the specification of the two options given in Section 2.1.1., we can obtain Eqs (12) and (13):

$$y_{1}=x_{1}+x_{2},\;\mathrm{and}$$ (12)

$$y_{2}=x_{2}.$$ (13)

Eq (12) indicates that the number of PT Operator 1’s passengers equals the total number of passengers taking Options 1 and 2, as it is the common trunk service between the origin and destination. Eq (13) holds per the definition of Option 2.

Let *C*_*i*_(*y*_*i*_) be the operating cost for the PT Operator *i*,∀*i* = 1,2 and suppose that it is a function continuously differentiable with respect to *y*_*i*_, and *dC*_*i*_(*y*_*i*_)/*y*_*i*_≥0, meaning that it does not decrease in *y*_*i*_. In this paper, *C*_*i*_(*y*_*i*_) is specified as,

$$C_{i}(y_{i})=\gamma_{i}+\mu y_{i}D_{i},\forall i=1,2,$$ (14)

where *γ*_*i*_ is the fixed cost associated with the operator *i*, and where *μ* is the constant cost of the PT operator per km per passenger.

Moreover, the profit function of PT Operator *i* is defined by passenger ticket revenue and operating costs:

$$\pi_{i}(y_{i})=p_{i}y_{i}-C_{i}(y_{i})$$ (15)

Then, social welfare can be measured by the sum of total profits and consumer surplus:

$$\mathrm{SW}=\mathrm{CS}+\pi_{1}+\pi_{2}$$ (16)

## 3 Market equilibria analysis

The focus will be on the fare and demand under five scenarios. Fare, demand, profit, and social welfare under collusion, social welfare maximizing, Bertrand, welfare maximizing and profit-maximizing Operator 1, and welfare maximizing and profit-maximizing Operator 2 are denoted by superscripts COLL, SW, B, SW-Op1, and SW-Op2, respectively.

### 3.1 Scenario I: Total profit-maximizing of Operator 1 and Operator 2

When the two operators are private enterprises and maximize total profit, the profit maximization problem for the two operators can be formulated as

$$max\prod^{COLL}=\prod_{1}^{COLL}+\prod_{2}^{COLL}$$ (17)

where

$$\prod_{1}^{COLL}=p_{1}y_{1}-Cy_{1}=(-mx_{1}-gx_{2}-W)y_{1}-\gamma -\mu y_{1}D_{1}$$ (18)

$$\prod_{2}^{COLL}=p_{2}y_{2}-C(y_{2})=[R+H(\lambda ,t_{1},t_{2})+(m-g)(x_{1}-x_{2})]y_{2}-\gamma -\mu y_{2}D_{2}$$ (19)

From the first-order optimality condition of Eq (17), the equilibrium of the fare and demand can be obtained.


$$x_{1}^{COLL}=\frac{1}{2(m^{2}-g^{2})}[m(W+\mu D_{1})-g(W+\mu D_{1}+\mu D_{2}-H-R)]$$ (20)

$$x_{2}^{COLL}=\frac{1}{2(m^{2}-g^{2})}[m(W+\mu D_{1}+\mu D_{2}-H-R)-g(W+\mu D_{1})]$$ (21)

$$p_{1}^{COLL}=\frac{1}{2}(\mu D_{1}-W),$$ (22)

$$p_{2}^{COLL}=\frac{1}{2}(H+R+\mu D_{2}).$$ (23)

Then the expression of social welfare is as follows:

$$SW^{COLL}=\prod_{1}^{COLL}+\prod_{2}^{COLL}+CS(x_{1}^{COLL},x_{2}^{COLL})$$ (24)

### 3.2 Scenario II: Social welfare maximizing of PT Operator 1 and PT Operator 2

When the two operators are public enterprises, the PT operators achieve social welfare maximizing by selecting the number of passengers, and the objective function can be expressed as follows:

$$max\;SW=\prod_{1}^{SW}+\prod_{2}^{SW}+CS(p_{1}^{SW},p_{2}^{SW})$$ (25)

According to the corresponding first-order optimal conditions the equilibrium prices and demand can be obtained as follows:

$$p_{1}^{SW}=\mu D_{1}$$ (26)

$$p_{2}^{SW}=\mu D_{2}$$ (27)

$$x_{1}^{SW}=\frac{1}{g^{2}-m^{2}}[m(W+\mu D_{1})-g(W+\mu D_{1}+\mu D_{2}-H-R)]$$ (28)

$$x_{2}^{SW}=\frac{1}{g^{2}-m^{2}}[m(W+\mu D_{1}+\mu D_{2}-H-R)-g(W+\mu D_{1})]$$ (29)

**Remark 1:** *By comparing Eqs (20) and (28) and (21) and (29), we can obtain $x_{1}^{SW}=2x_{1}^{COLL}$, $x_{2}^{SW}=2x_{2}^{COLL}$. Hence, the number of passengers of the two options under SW-max is twice that of Collusion.*

### 3.3 Scenario III: Simultaneous competition on price (Bertrand)

When the two operators are private enterprises and maximize their profits in price competition, their objective functions can be modelled as follows:

$$\max\prod_{1}^{B}$$ (30)

$$\max\prod_{2}^{B}$$ (31)

According to the corresponding first-order optimal conditions the equilibrium price and demand can be obtained.


$$p_{1}^{B}=\frac{1}{g+7m}[m(H+R-3W+4\mu D_{1}-\mu D_{2})-gW]$$ (32)

$$p_{2}^{B}=\frac{1}{g+7m}[g(H+R+2W+2\mu D_{1})+m(3H+3R-2W+4\mu D_{2}-2\mu D_{1})]$$ (33)

$$\begin{array}{l} x_{1}^{B}=\frac{1}{\left(g^{2}-m^{2})(g+7m\right)}[m^{2}(H+R+4W+4\mu D_{1}-\mu D_{2})+2g^{2}(H+R+\mu D_{1})\\ \;+mg(9H+9R-4W-2\mu D_{1}-5\mu D_{2})] \end{array}$$ (34)

$$\begin{array}{l} x_{2}^{B}=\frac{1}{\left(g^{2}-m^{2})(g+7m\right)}[m^{2}(-9H-9R+6W+2\mu D_{1}-5\mu D_{2})\\ \;-mg(3H+3R+4W+6\mu D_{1}-\mu D_{2})] \end{array}$$ (35)

Social welfare can be described as follows:

$$SW^{B}=\prod_{1}^{B}+\prod_{2}^{B}+CS(p_{1}^{B},p_{2}^{B})$$ (36)

### 3.4 Scenario IV: Maximizing social welfare and the profit of PT Operator 1(SW-Op 1)

When PT Operator 1 is a private enterprise and competes in price to maximize its profit, and PT Operator 2 is a public enterprise aiming at maximizing social welfare, then PT Operators 1 and 2 will solve the following decision problems at the same time:

$$\underset{p_{1}^{SW-Op1}}{max}\prod_{1}^{SW-Op1}$$ (37)

$$\underset{p_{2}^{SW-Op1}}{max}\begin{array}{c} SW= \end{array}\prod_{1}^{SW-Op1}+\prod_{2}^{SW-Op1}+CS(p_{1}^{SW-Op1},p_{2}^{SW-Op1})$$ (38)

Then the equilibrium price and demand can be obtained as follows:

$$p_{1}^{SW-Op1}=\frac{g\mu D_{1}+m(\mu D_{1}-\mu D_{2}+H+R-2W)}{3m+g}$$ (39)

$$p_{2}^{SW-Op1}=\frac{g(H+R-2W-2\mu D_{1})+m(2\mu D_{1}+4\mu D_{2}-H-R+2W)}{3m+g}$$ (40)

$$\begin{array}{l} x_{1}^{SW-Op1}=\frac{1}{\left(g^{2}-m^{2})(3m+g\right)}\{m^{2}(\mu D_{1}+\mu D_{2}+H+R+W)\\ \;+g^{2}(W+\mu D_{1})+mg(-2\mu D_{1}-3\mu D_{2}-2W+3H+3R)\} \end{array}$$ (41)

$$\begin{array}{l} x_{2}^{SW-Op1}=\frac{1}{\left(g^{2}-m^{2})(3m+g\right)}\{3m^{2}(\mu D_{1}+\mu D_{2}+W-H-R)\\ \;-g^{2}(\mu D_{1}+W)-mg(2W+2\mu D_{1}+\mu D_{2}-H-R)\} \end{array}$$ (42)

### 3.5 Scenario V: Maximizing social welfare and the profit of Operator 2 (SW-Op 2)

When PT Operator 2 is a private enterprise and competes in price to maximize its profit, and PT Operator 1 is a public enterprise aimed at maximizing social welfare, then Operators 1 and 2 solve the following decision problems at the same time:

$$\underset{p_{1}^{SW-Op2}}{max}SW=\prod_{1}^{SW-Op2}+\prod_{2}^{SW-Op2}+CS(p_{1}^{SW-Op2},p_{2}^{SW-Op2})$$ (43)

$$\underset{p_{2}^{SW-Op2}}{max}\prod_{2}^{SW-Op2}$$ (44)

Then the equilibrium price and demand can be obtained as follows:

$$p_{1}^{SW-Op2}=\frac{m(4\mu D_{1}+\mu D_{2}-H-R+W)-gW}{3m+g},$$ (45)

$$p_{2}^{SW-Op2}=\frac{g(2\mu D_{1}+\mu D_{2}+2W)-m(2\mu D_{1}-\mu D_{2}-2H-2R+2W)}{3m+g}$$ (46)

$$\begin{array}{l} x_{1}^{SW-Op2}=\frac{1}{\left(g^{2}-m^{2})(3m+g\right)}\{m^{2}(4\mu D_{1}+\mu D_{2}-H-R+4W)\\ \;-2mg(\mu D_{1}+\mu D_{2}+W-H-R)+g^{2}(2\mu D_{1}+\mu D_{2}+H+R)\}, \end{array}$$ (47)

$$x_{2}^{SW-Op2}=\frac{2m^{2}(\mu D_{1}+\mu D_{2}+W-H-R)-2mg(3\mu D_{1}+2\mu D_{2}+2W)}{\left(g^{2}-m^{2})(3m+g\right)}.$$ (48)

## 4. Main theorems about equilibria

In this section, we delve into nine pivotal theorems that elucidate the dynamics of demand, fare, social welfare, and profitability under the different scenarios. We focus on the impact of the implementation of discount incentives on demand, fare, social welfare, and profitability.

**Theorem 1.** In all the scenarios but scenario V, offering discounts by PT Operator 2 results in an increase in demand for Option 2, and concurrently, there is a decrease in demand for Option 1 when *m*^2^−*g*^2^>0, *g*∈(0,1).

**Proof.** The first-order differentiation of demand with respect to discounts under the base case and other scenarios is as follows:

Base case: $\frac{\partial x_{1}}{\partial H}=\frac{g}{g^{2}-m^{2}}<0$, $\frac{\partial x_{2}}{\partial H}=\frac{-m}{g^{2}-m^{2}}>0$,

Collusion: $\frac{\partial x_{1}^{COLL}}{\partial H}=\frac{g}{2(m^{2}-g^{2})}<0$, $\frac{\partial x_{2}^{COLL}}{\partial H}=\frac{-m}{2(m^{2}-g^{2})}>0$,

SW-Max: $\frac{\partial x_{1}^{SW}}{\partial H}=\frac{g}{g^{2}-m^{2}}<0$, $\frac{\partial x_{2}^{SW}}{\partial H}=\frac{-m}{g^{2}-m^{2}}>0$,

Bertrand: $\frac{\partial x_{1}^{B}}{\partial H}=\frac{m^{2}+9mg+2g^{2}}{\left(g^{2}-m^{2})(g+7m\right)}<0$, $\frac{\partial x_{2}^{B}}{\partial H}=\frac{-9m^{2}-3mg}{\left(g^{2}-m^{2})(g+7m\right)}>0$,

SW-Op 1: $\frac{\partial x_{1}^{SW-Op1}}{\partial H}=\frac{m^{2}+3mg}{\left(g^{2}-m^{2})(3m+g\right)}<0$, $\frac{\partial x_{2}^{SW-Op1}}{\partial H}=\frac{-3m^{2}+mg}{\left(g^{2}-m^{2})(3m+g\right)}>0$,

SW-Op 2: $\frac{\partial x_{1}^{SW-Op2}}{\partial H}=\frac{-m^{2}+2mg+g^{2.}}{\left(g^{2}-m^{2})(3m+g\right)}$, $\frac{\partial x_{2}^{SW-Op1}}{\partial H}=\frac{-2m^{2}}{\left(g^{2}-m^{2})(3m+g\right)}>0$, under *m*^2^−*g*^2^>0, *g*∈(0,1).

In essence, the introduction of a discount by PT Operator 2 influences consumer behavior in the transportation market. When PT Operator 2 offers a discount on their services, it becomes more attractive to potential passengers because of the reduced cost. This competitive pricing strategy is likely to draw more customers away from other transport options, including those provided by a third-party service. As a result, the demand for PT Operator 2’s services is expected to rise, as more individuals opt for their discounted fares over other, potentially more expensive, alternatives. This increase in demand can be attributed to several factors. First, the discount makes PT Operator 2’s services more affordable, which is a significant consideration for many consumers, especially those who are budget conscious. Second, the perception of value is enhanced when a service is offered at a lower price, which can lead to increased customer satisfaction and loyalty.

**Theorem 2**. The price of PT Operator 1 is independent of the discount under Collusion and SW-Max, increases under Bertrand, and SW-Op 1 and decrease under SW-Op 2. The price of PT Operator 2 is independent of the discount under SW-Max, increasing under Collusion, Bertrand, and SW-Op 2 and increases under SW-Op 1.

**Proof.** The method for the proof is very simple and therefore the detailed proof is not reported here.

According to **Theorem 2,** the impact of discounts on ticket prices in different scenarios is uniquely determined. This helps Operators adapt to changes in ticket prices and develop feasible ticket prices, thereby enhancing the competitiveness of transportation services.

**Theorem 3**. *Under Collusion*, *optimal total profits can be achieved when m*^2^−*g*^2^>0, *g*∈(0,1).

**Proof.** According to Eq (15), we can obtain the first-order differentiation of total profits with respect to the discount as follows:

$\frac{\partial (\prod_{1}^{COLL}+\prod_{2}^{COLL})}{\partial H}=\frac{1}{4(m^{2}-g^{2})}[m(3H+3R-3\mu D_{1}-3\mu D_{2}-W)+g(3\mu D_{1}+3W)]$, and the second-order differentiation of total profits with respect to discount as follows: $\frac{\partial^{2}(\prod_{1}^{COLL}+\prod_{2}^{COLL})}{\partial H^{2}}=\frac{3m}{4(m^{2}-g^{2})}>0$. Hence, we conclude that the total profit function is a strictly concave function with a maximum, and the optimal total profits can be achieved.

**Theorem 3** states that when two Operators collude with each other, there is a most favourable discount that maximizes their collective profits rather than competing with each other. This approach has pricing reference value for operators, as it can attract passengers and achieve operational goals.

**Theorem 4.** *Under Bertrand*, *the optimal profit of PT Operator 2 can be achieved when m*^2^−*g*^2^>0, *g*∈(0,1).

**Proof.** According to the profit expression of PT Operator 2, the first- differentiation with respect to the discount is as follows:

$$\begin{array}{l} \frac{\partial \pi_{2}^{B}}{\partial H}=\frac{-3m(3m+g)}{\left(g^{2}-m^{2})(g+7m\right)}\{\frac{1}{g+7m}[g{\left(H+R+2W+2\mu D\right)}_{1}\\ \;+m(3H+3R-2W+4\mu D_{2}-2\mu D_{1})]-\mu D_{2}\}\\ \;+\frac{3m+g}{(g^{2}-m^{2}){\left(g+7m\right)}^{2}}[m^{2}(-9H-9R+6W+2\mu D_{1}-5\mu D_{2})\\ \;-mg(3H+3R+4W+6\mu D_{1}-\mu D_{2})] \end{array}$$

Then, the second-differentiation with respect to the discount is as follows:

$$\frac{\partial^{2}\pi_{2}^{B}}{\partial H^{2}}=\frac{-6m{\left(3m+g\right)}^{2}}{(g^{2}-m^{2}){\left(g+7m\right)}^{2}}>0,\;if\;m>g,g\in (0,1).$$

Therefore, the profit function of Operator 2 is strictly concave and can always yield the optimal profit.

**Theorem 5.** *Under SW-Op 1*, *the profits of PT Operator 1 will increase with the increasing of discounts*.

**Proof.** Considering the differentiation of the profits of PT Operator 1 with respect to the discount under SW-Op 1: $\frac{\partial \prod_{1}^{SW-Op1}}{\partial H}=\frac{p_{1}-\mu D_{1}}{m+g}>0$, the profit of PT Operator 1 monotonically increases, meaning that the larger the discount is for PT Operator 2, the greater the profit for PT Operator 1.□

**Theorem 6**. *Under Collusion*, *social welfare increases with increasing discounts*.

**Proof.** According to Eq (24), we can obtain the first differentiation of social welfare with respect to the discounts as follows:

$$\frac{\partial (SW^{\mathrm{COLL}})}{\partial H}=\frac{\partial (\prod_{1}^{COLL}+\prod_{2}^{COLL}+CS(x_{1}^{COLL},x_{2}^{COLL}))}{\partial H}=x_{2}^{COLL}>0.$$

Therefore, we confirm that social welfare monotonically increases with discounts.

**Theorem 7.** *Under SW—Max*, *the optimal social welfare can be achieved when m*^2^−*g*^2^>0, *g*∈(0,1).

**Proof.** According to the expression of the first- differentiation of social welfare with respect to the discount

$$\begin{array}{c} \frac{\partial (\prod_{1}^{SW}+\prod_{2}^{SW}+CS(p_{1},p_{2}))}{\partial H}=\frac{1}{m+g}(p_{1}-\mu D_{1})+\frac{m(p_{2}-\mu D_{2})}{g^{2}-m^{2}}+\frac{g(p_{1}+W)}{\left(m^{2}-g^{2}\right)}\\ -\frac{2m(p_{1}+p_{2}+W-H(\lambda ,t_{1},t_{2})-R)}{2(m^{2}-g^{2})} \end{array},$$

and the second differentiation

$$\frac{\partial^{2}(\prod_{1}^{SW}+\prod_{2}^{SW}+CS(p_{1},p_{2}))}{\partial H^{2}}=\frac{m}{\left(m^{2}-g^{2}\right)}>0.$$

Therefore, we conclude that the social welfare function is strictly concave and can always yield the optimal social welfare.

**Theorem 8**. *Under Bertrand*, *the optimal social welfare is achievable*.

**Proof.** Similar to that for **Theorem 7**

**Theorem 9.** *Under SW-Op 2*, *the optimal social welfare and PT Operator’s 2 profit can be achieved*.

**Proof.** Similar to that for **Theorem 4** and **Theorem 7.**

**Theorems 4–9** explain the changes in the profits and social welfare of operators under the different scenarios. With discount incentives, Operator 2 is more likely to achieve optimal profits (**Theorems 4 and 8**). This is because the shift in passenger preferences towards PT Operator 2 may have a broader impact on the transportation market, leading to an increase in the PT Operator’s market share. Although social welfare increases correspondingly with the increasing of discounts (**Theorem 6**), third-party services may need to reconsider their pricing strategies to maintain competitiveness, which may trigger price wars or prompt other operators to launch discounts or promotional activities to retain or attract passengers.

The proof of our theorems rigorously follows the analysis for ticket pricing, demand, social welfare assessment, and profit determination used by Clark et al [20]. and Wang et al. [35]. Our focus diverges from the literature emphasizing factors such as distance, speed, and frequency. Instead, this paper highlights the pivotal role of discount incentives in consumer behaviour and market outcomes. Echoing the insights of Wang et al. [33] within the realm of quantity competition, our study reveals that social welfare reaches its zenith under scenarios of price competition and collusion, akin to their findings.

## 5. Numerical simulation

We will conduct numerical simulations for each scenario and verify the theorem mentioned in Section 4. The theorem can be more intuitively understood from the simulation result graph. For each scenario, we pay special attention to the impact of discounts on ticket prices, profits, and social welfare. In addition, we will further investigate the impact of changes in operator service time on optimal profits and social welfare. The parameters used in the test are shown in Table 2. The travel time [*t*_1_,*t*_2_,*t*_3_] represents the service time of PT Operators 1, 2, and potentially, the 3^rd^ party operator.

**Table 2:** Parameters used in the test.

Variables	Scenario					
	Base case	I	II	III	IV	V
*a*_1_	160	225	225	250	230	190
*a*_2_	170	240	240	290	230	220
*b*	/	1	1	1	0.1	1
*m*	1	1	1	1	0.1	0.1
*g*	0.5	0.5	0.5	0.5	0.1	1
*γ*_1_	40	45	45	45	45	45
*γ*_2_	30	40	40	40	40	40
*μ*	1	1	1	1	10	10
*D*_1_	18	18	18	18	10	10
*D*_2_	4.5	4.5	4.5	4.5	5	5

### 5.1 Demand and discounts

**Fig 2:**
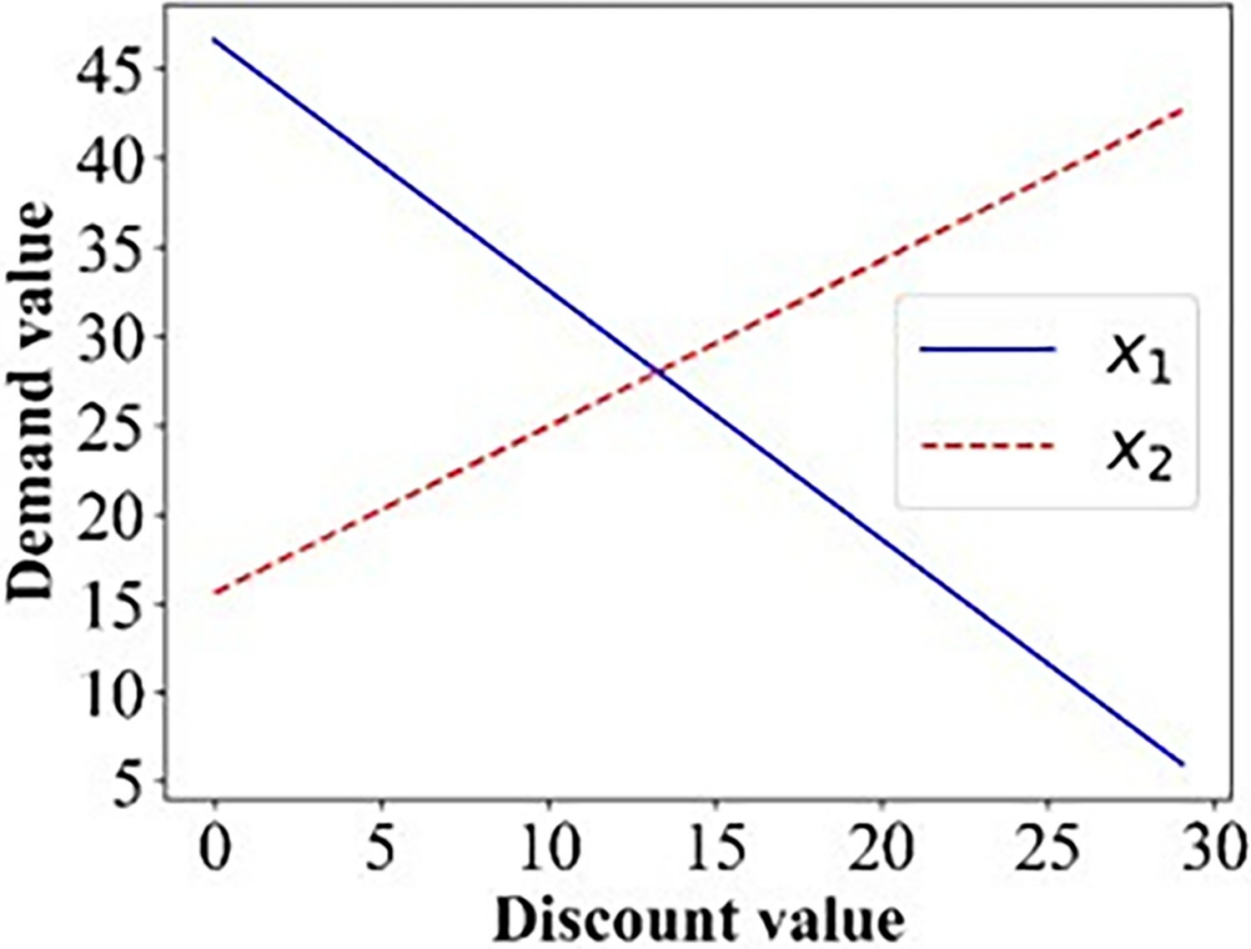
The demand for the two options in the base case.

**Fig 3:**
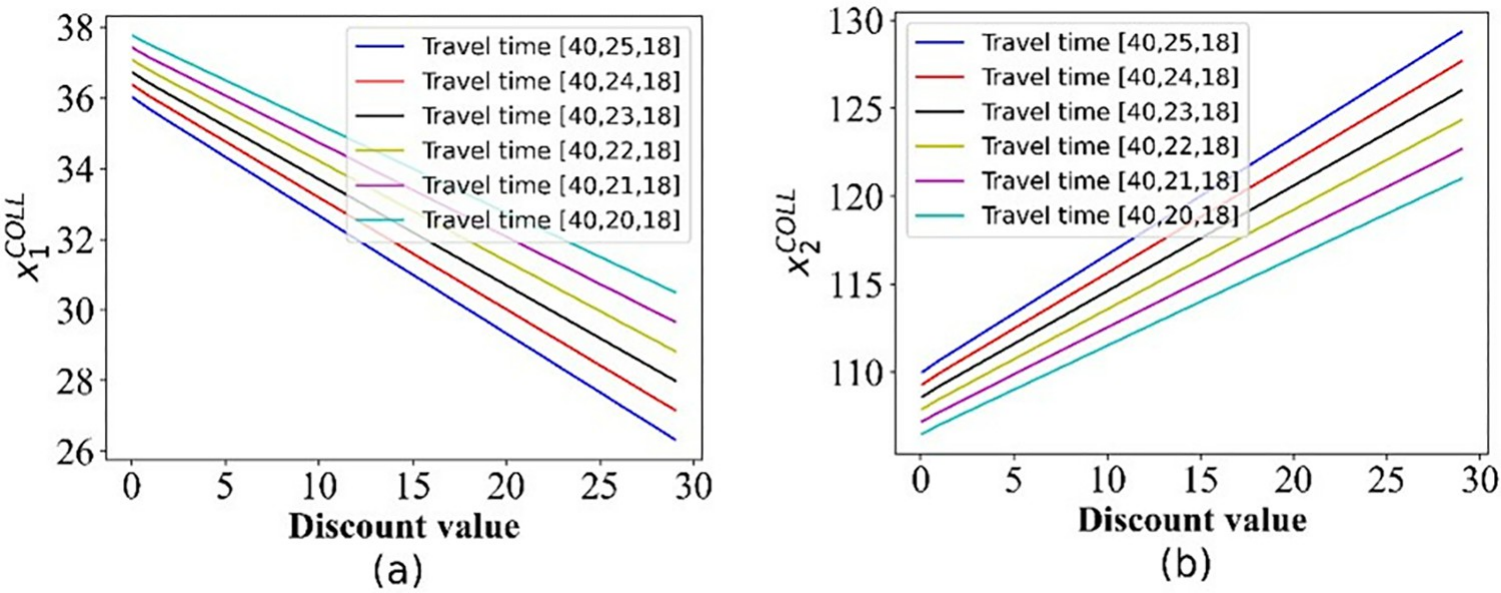
The demand for the two options in scenario I. (a) Option 1; (b) Option 2.

**Fig 4:**
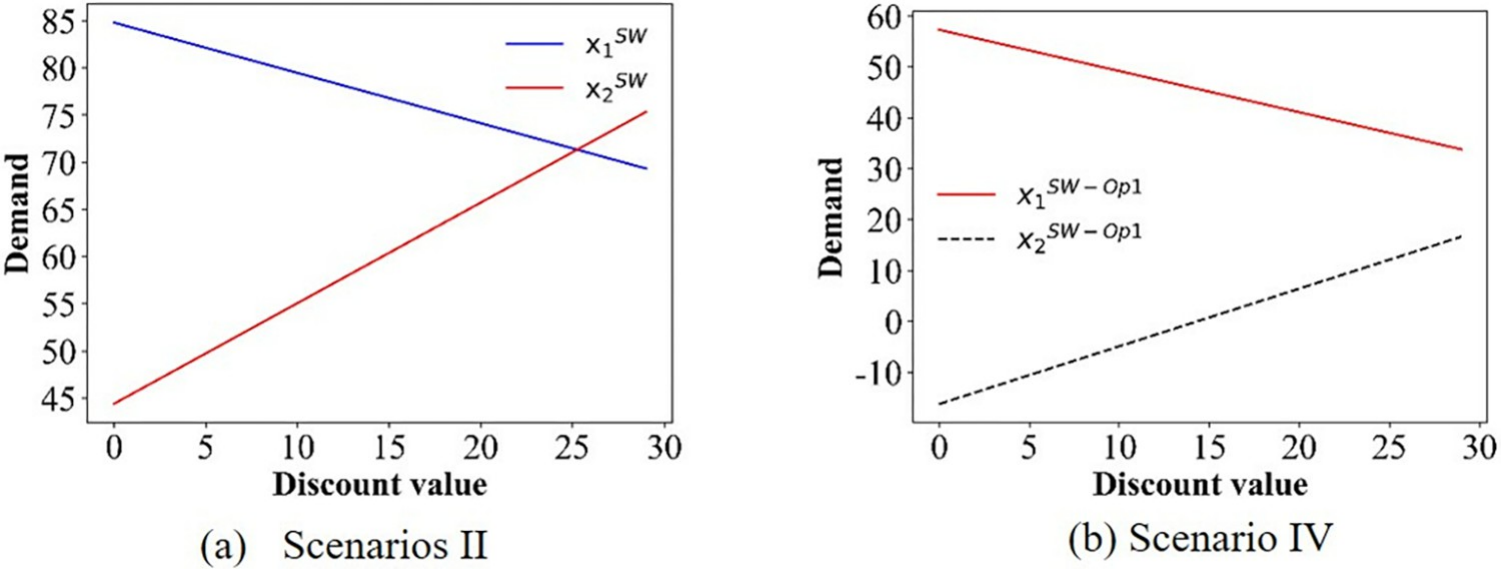
The demand for the two options in scenarios II and IV.

**Fig 5:**
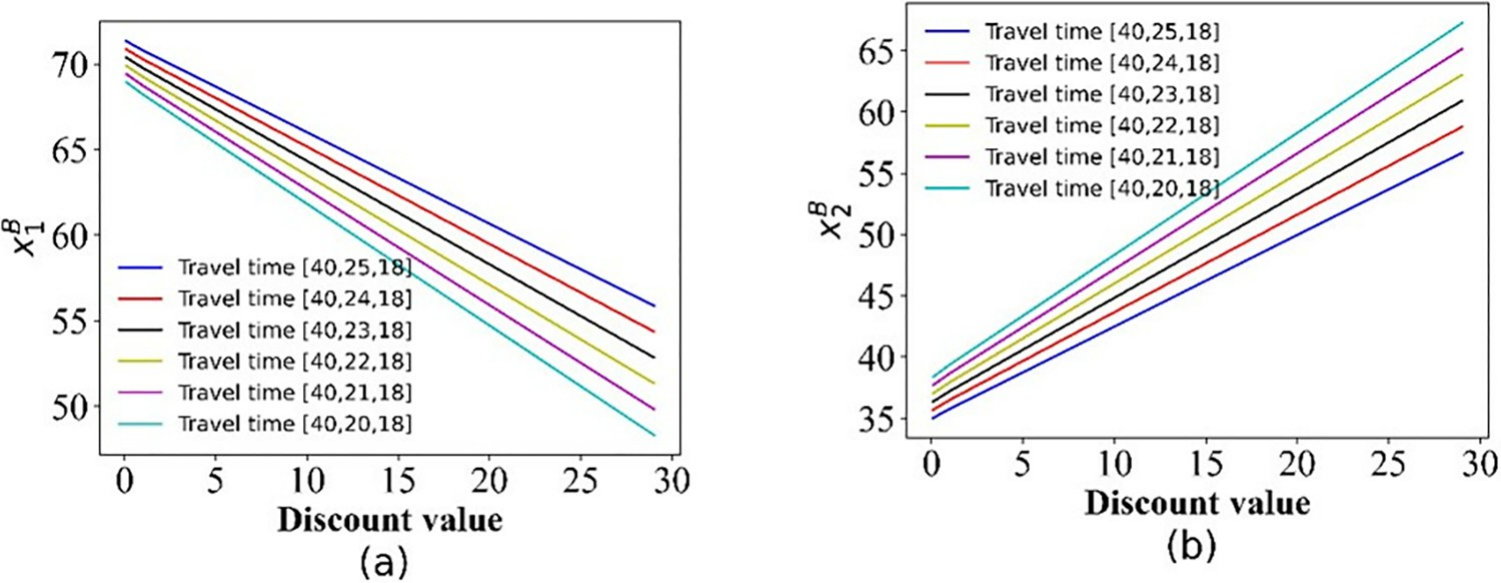
The demand for the two options in scenario III. (a) Option 1; (b) Option 2.

On the basis of the formulation in Sections 3 we resort to numerical studies to illustrate the properties. The effect of ticket discounts on the demand for the two options is shown in Figs 2–5.

As expected, Figs 2–5 show that with the increase in the discount, the number of passengers changes in the opposite direction of the two options, the demand for Operator 2 gradually increases, whereas that for the 3^rd^ party gradually decreases.

### 5.2 Price and discounts

**Fig 6:**
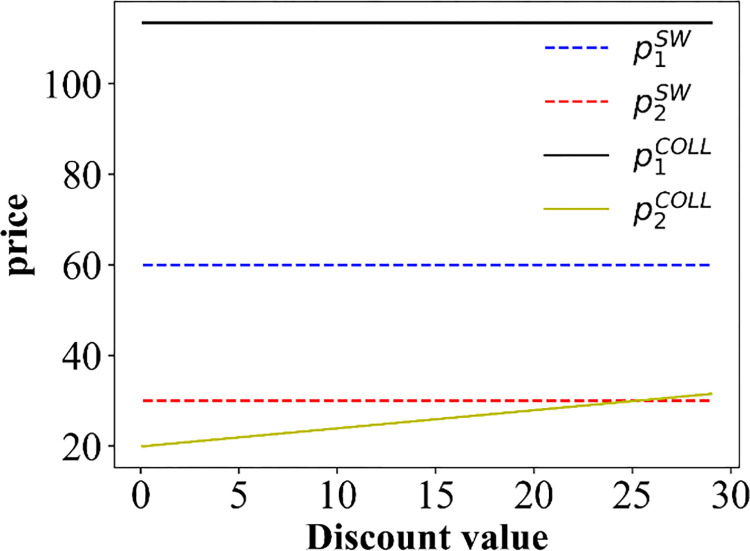
The price curve varies with discounts in scenarios I and II.

**Fig 7:**
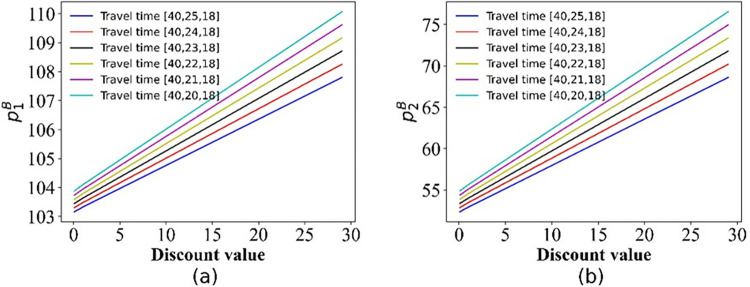
The price curve varies with discounts in scenario III. (a) PT Operator 1; (b) PT Operator 2.

**Fig 8:**
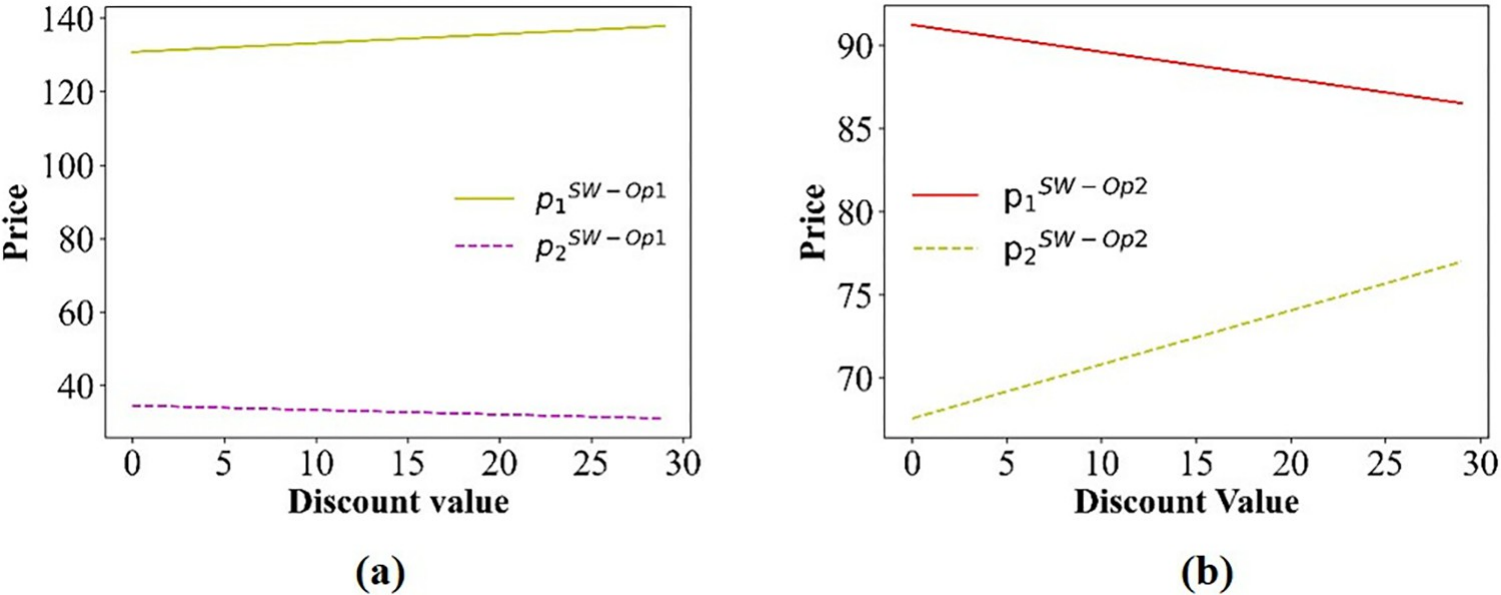
The price curve varies with discounts. (a) scenario IV; (b) scenario V.

For this section, we pay special attention to the impact of discounts on ticket prices. The numerical simulation results regarding the change in price are shown in Figs 6–8.

As depicted in Fig 6, there is no variation in the price charged by PT Operator 1, which is attributed to the fact that only passengers opting for PT Operator 2 are eligible for discounts. In addition, we can obtain $p_{1}^{COLL}>p_{1}^{SW}>p_{2}^{COLL}$. The change in prices only depends on the transportation distance covered by the operators. From Fig 7, we can see that with increasing discounts, the prices of PT Operators 1 and 2 are increase, and the price of Operator 1 is much higher than that of Operator 2. This gives the same conclusion in **Theorem 2**. Moreover, the travel time also affects the price. Specifically, decreasing the travel time (improving the operation speed) of PT Operator 2 increases the slope of the price curve ($p_{1}^{B}$ and $p_{2}^{B}$), indicating that the price increases faster. That is, the travel time accelerates the growth rate of prices. For scenario IV, we also see more clearly that the price of PT Operator 2 decreases with the discount, but the ticket price of PT Operator 1 increases as the discount increases (Fig 8A). However, in scenario V, the ticket price varies with the discount (Fig 8B), which is the opposite that of scenario IV.

### 5.3 Profits and discounts

**Fig 9:**
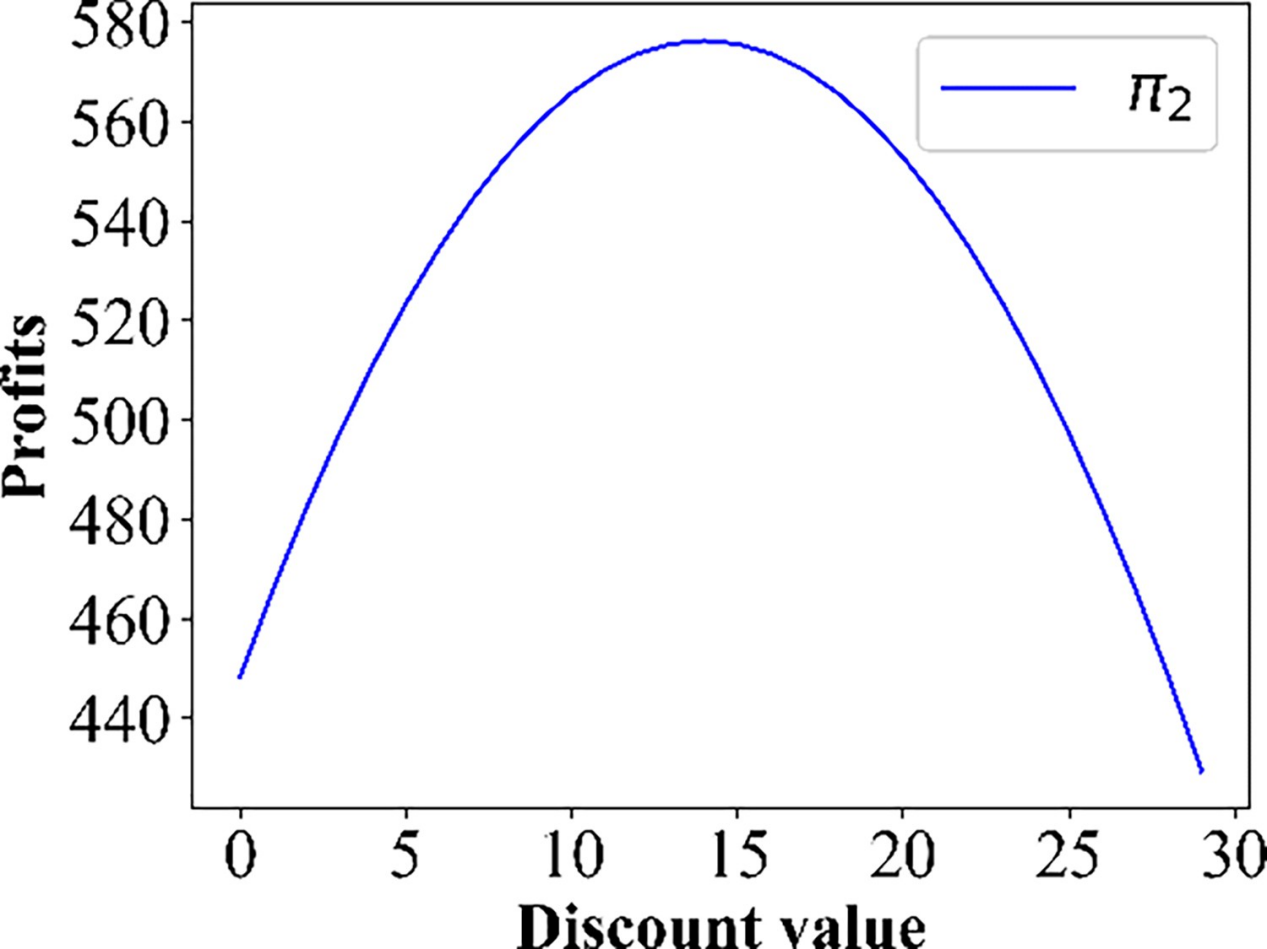
Change in the profits of PT Operator 2 in the base case.

**Fig 10:**
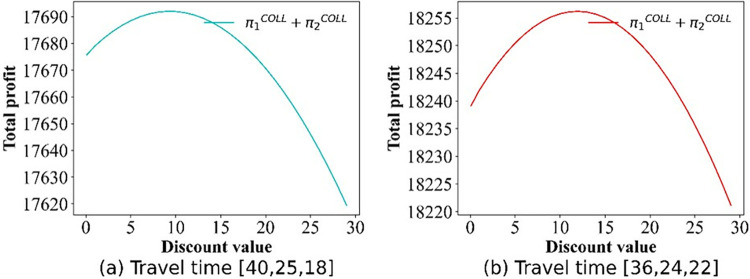
Effect of the discounts on the operator’s total profit.

**Fig 11:**
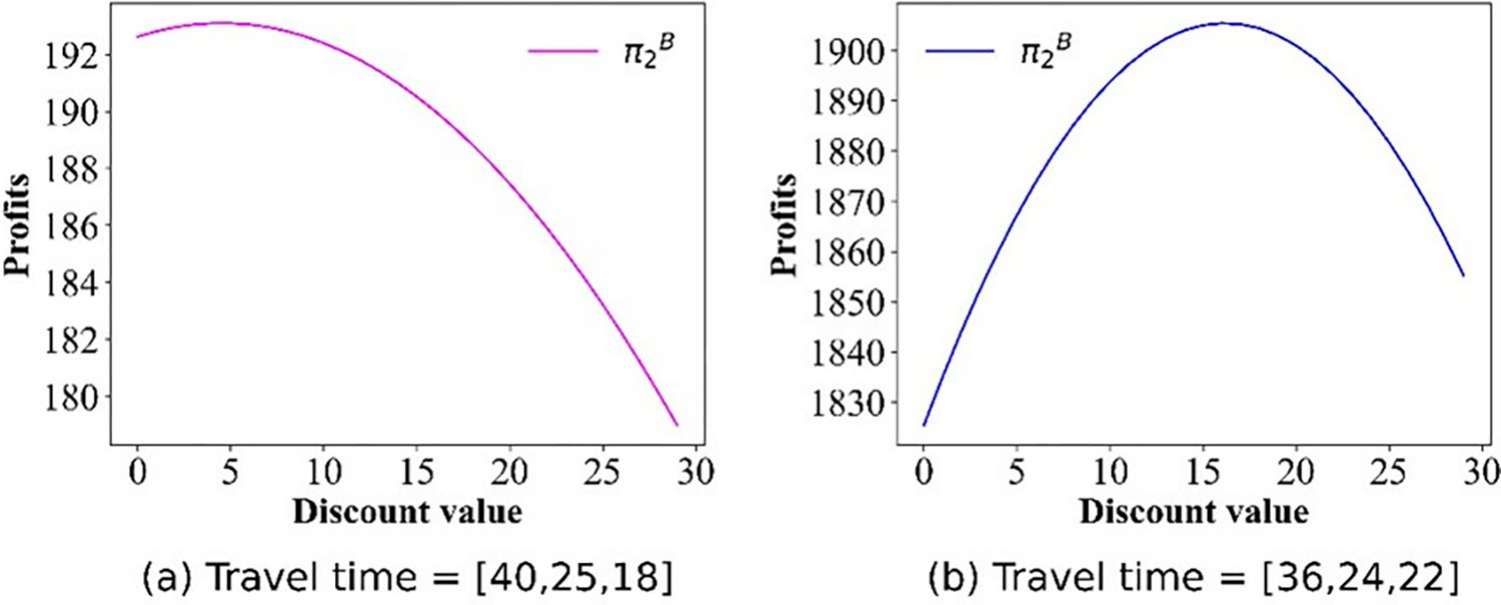
Effect of the discounts on the profit curve of Operator 2 in scenario III.

**Fig 12:**
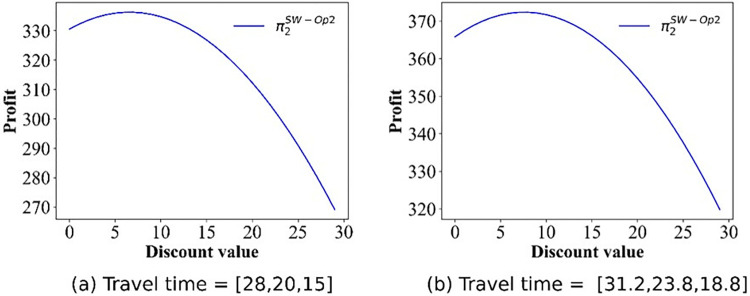
Effect of the discounts on the profits of Operator 2 in scenario V.

For this section, we pay attention to the impact of discounts on profits. The numerical simulation results regarding the change in price are shown in Figs 9–12. Owing to the negative profit for Operator 2 (Operator 1) in scenario IV(V), we do not present the findings for this scenario. Figs 9–12, confirm that in all other scenarios, there exists an optimal discount that ensures the actual maximization of Operator 2’s profit or total profit.

### 5.4 Social welfare and discounts

**Fig 13:**
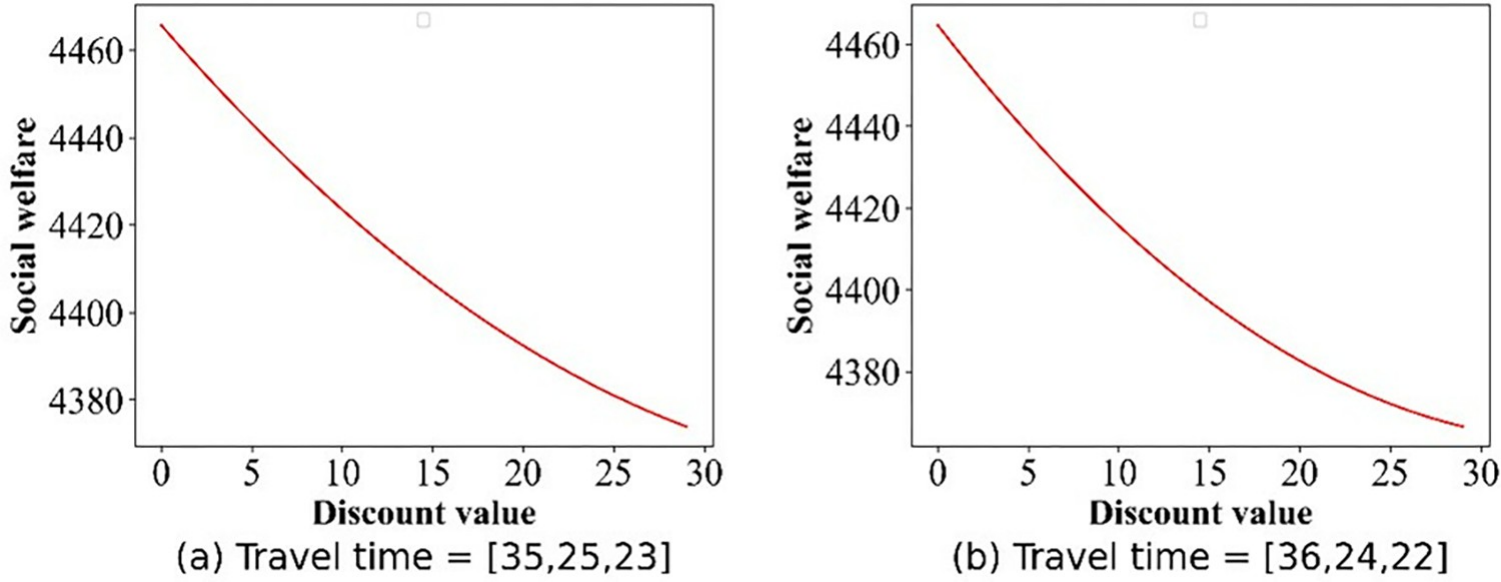
Effect of the discounts on social welfare in the base case.

**Fig 14:**
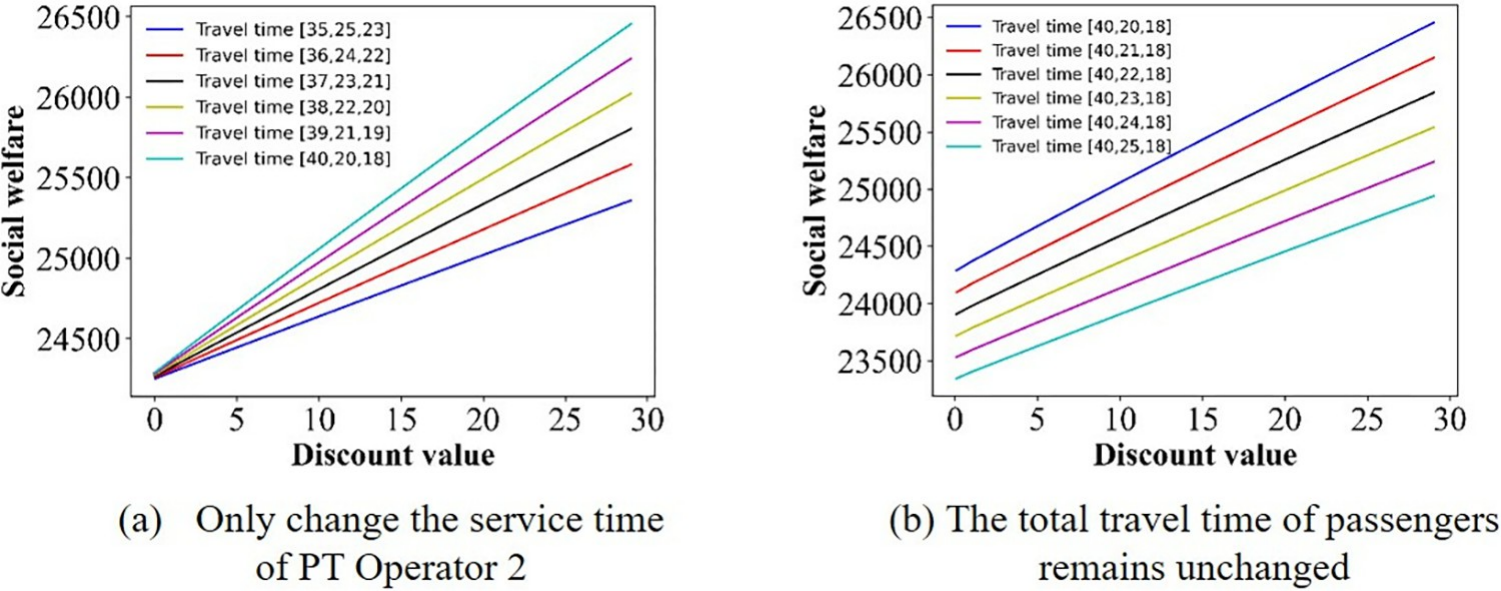
Effect of the discounts on social welfare in scenario I.

**Fig 15:**
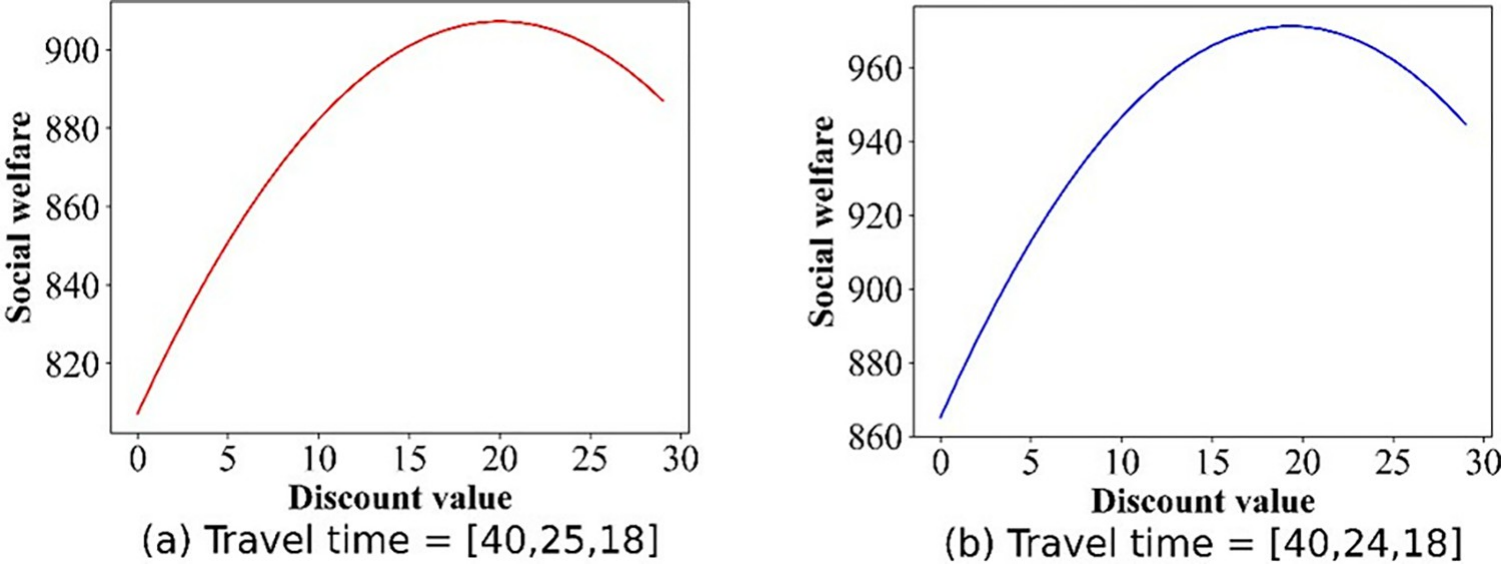
Effect of the discounts on social welfare in scenario II.

**Fig 16:**
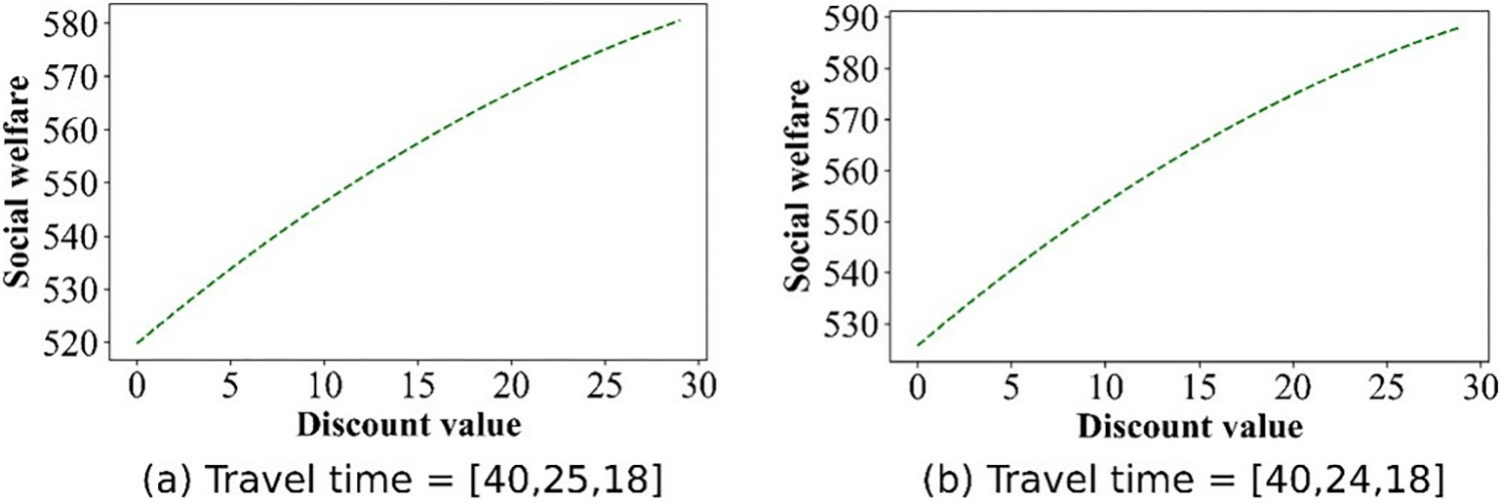
Effect of discounts on social welfare in scenario III.

**Fig 17:**
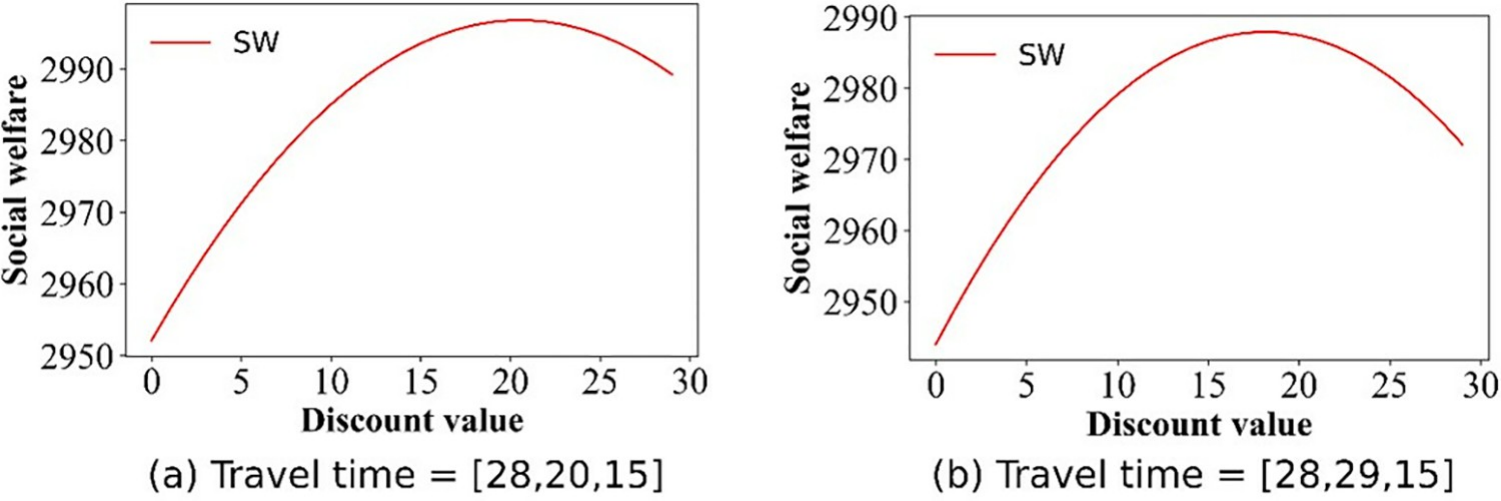
Effect of the discounts on social welfare in scenario V.

In this section, we focus on examining the impact of discount policies on social welfare, elucidating how these incentives can influence overall societal benefits. The numerical simulation outcomes detailing the social welfare fluctuations are presented in Figs 13–17. From Fig 13, we can deduce that, in the base case, social welfare attains its minimum threshold, a finding that corroborates the principles outlined in Theorem 1. Fig 14 illustrates the variation in social welfare in response to discounts within Scenario II. Unlike total profit, social welfare positively correlated with the magnitude of discounts, a trend that aligns precisely with the predictions of **Theorem 6.**

Figs 15–17 show that for Scenarios II, III, and V, the optimal social welfare is achievable, but the corresponding discounts do not align with those corresponding to the optimal profit. Taking Scenario III as an example, Tables 3 and 4 present the discounts corresponding to the maximum social profit under different service time combinations. The variation in discounts is not linear.

**Table 3:** The discounts and social welfare corresponding to Operator 2’s maximum profit when the service time of Operator 2 varies.

Category	Travel time of two PT operators and the 3^rd^ party					
	[40,25,18]	[40,24,18]	[40,23,18]	[40,22,18]	[40,21,18]	[40,20,18]
*H*(*λ*,*t*_1_,*t*_2_)	15.75	16.00	17.00	17.10	18.05	18.00
$x_{1}^{SW}$	75.50	74.67	73.33	72.60	71.30	70.67
$x_{2}^{SW}$	63.00	64.67	67.33	68.80	71.40	72.67
SW	907.25	971.22	1037.22	1105.24	1175.21	1247.22

**Table 4:** The discounts and social welfare correspond to Operator 2’s maximum profit when the total travel time remains unchanged.

Category	Travel time of two PT operators and the 3^rd^ party					
	[35,30,23]	[36,29,22]	[37,28,21]	[38,27,20]	[39,26,19]	[40,25,18]
*H*(*λ*,*t*_1_,*t*_2_)	7.50	10.50	13.50	15.95	15.60	15.75
$x_{1}^{SW}$	81.00	79.00	77.00	75.37	75.60	75.50
$x_{2}^{SW}$	52.00	56.00	60.00	63.27	62.80	63.00
SW	877.00	895.00	905.00	907.23	907.24	907.25

### 5.5. Distance and discounts

This section delves into the correlation between discounts and service distances. We scrutinize these relationships through differentiation discounts with respect to the distance for the services provided by the PT Operator and the competing operator. For ease of presentation, we adopt the superscript superscripts COLL, B, SW-Op1, and SW-Op2 to differentiate discounts under the different scenarios.

According to Eqs (20), (32), (39) and (45), we can obtain the following formulas:

$$H^{COLL}=W+\mu D_{1}+\mu D_{2}-R-\frac{m(W+\mu D_{1})-2(g^{2}-m^{2})x_{1}^{COLL}}{g}$$ (49)

$$H^{B}=\frac{gW+2(g+7m)p_{1}^{B}}{m}+3W-4\mu D_{1}+\mu D_{2}-R$$ (50)

$$H^{SW-Op1}=\frac{p_{1}^{SW-Op1}(3m+g)-g\mu D_{1}-m(\mu D_{1}-\mu D_{2}+R-2W)}{m}$$ (51)

$$H^{SW-Op2}=\frac{m(4\mu D_{1}+\mu D_{2}-R+W)-gW-(3m+g)p_{1}^{SW-Op2}}{m}$$ (52)

Furthermore, we calculate the derivatives of the discount in relation to the distance served by both PT Operators 1 and 2 as follows:

$$\frac{\partial H^{COLL}}{\partial D_{1}}=\frac{\mu (g-m)}{g}<0,\;\frac{\partial H^{B}}{\partial D_{1}}=-4\mu <0,\;\frac{\partial H^{SW-Op1}}{\partial D_{1}}=\frac{-\mu (m+g)}{m}<0$$

$$\frac{\partial H^{SW-Op2}}{\partial D_{1}}=4\mu >0,\;\frac{\partial H^{COLL}}{\partial D_{2}}=\frac{\partial H^{B}}{\partial D_{2}}=\frac{\partial H^{SW-Op1}}{\partial D_{2}}=\frac{\partial H^{SW-Op2}}{\partial D_{2}}=\mu >0.$$

In scenarios I, III, and IV, it becomes clear that the discount consistently diminishes as the service distance of PT Operator 1 extends. Conversely, in scenario V, an intriguing reversal is observed: the discount escalates with an increase in PT Operator 1’s service distance. Uniformly across all the scenarios, an increase in the service distance for PT Operator 2 is correlated with an increase in the discount. This observation aligns with our foundational hypothesis: the more extensive the service distance offered by Operator 2 is, the more substantial the discount provided. This correlation underscores a direct relationship between the scope of service and incentivizing discounts within the public transport domain. In scenario II, we find that the operator’s ticket price is solely related to its service distance (see Eqs (28) and (29)). Therefore, the discount is unrelated to their distance.

## 6. Conclusions

In this paper, we have developed a trimodal public transportation competition model between an OD pair, with one operator employing ticket price discounts as an incentive to attract more passengers. On this basis, we studied five different competitive scenarios, from which we deduced the equilibrium ticket pricing and demand levels for each case. Theoretical analysis and numerical simulations have demonstrated that discounts effectively lure an increased number of passengers and contribute to the realization of optimal profits. In addition, our findings revealed that when the maximization of social welfare is prioritized, the passenger count nearly doubles compared with the scenarios focused solely on profit maximization. This observation underscores the significant impact of operational objectives on passenger attraction and highlights the potential for more equitable outcomes when social welfare is the primary consideration. Nonetheless, the precise dynamics of how the optimal total profit, social welfare, and their corresponding optimal discount fluctuate in response to variations in travel times across operators remain elusive. This complexity underscores the need for further investigation into the nuanced interplay between pricing strategies, service timing, and their broader economic and societal implications.
